# Determinants of adherence and effects on health-related quality of life after myocardial infarction: a prospective cohort study

**DOI:** 10.1186/s12877-018-0827-y

**Published:** 2018-06-07

**Authors:** Gundula Krack, Rolf Holle, Inge Kirchberger, Bernhard Kuch, Ute Amann, Hildegard Seidl

**Affiliations:** 1 Munich Center of Health Sciences (MC-Health), Institute for Health Economics and Management, Ludwig-Maximilians-Universität München, Ludwigstr. 28 RG, 80539 Munich, Germany; 2 Helmholtz Zentrum München, Institute of Health Economics and Health Care Management, Neuherberg, Germany; 3 UNIKA-T Augsburg, Chair of Epidemiology, Ludwig-Maximilians Universität München, Augsburg, Germany; 4 Helmholtz Zentrum München, Institute of Epidemiology II, Neuherberg, Germany; 5 Central Hospital of Augsburg, MONICA/KORA Myocardial Infarction Registry, Augsburg, Germany; 6 Hospital of Nördlingen, Department of Internal Medicine/Cardiology, Nördlingen, Germany

**Keywords:** Myocardial infarction, Coronary artery disease, Aged, Healthy lifestyle, Patient reported outcome measures, Nurses, Case management, Adherence, Quality of life

## Abstract

**Background:**

Adherence to recommendations and medication is deemed to be important for effectiveness of case management interventions. Thus, reasons for non-adherence and effects on health-related quality of life (HRQoL) should be fully understood. The objective of this research was to identify determinants of non-adherence to medication and recommendations, and to test whether increased adherence improved HRQoL in patients after myocardial infarction (MI) in a case management intervention.

**Methods:**

Data were obtained from the intervention group of the KORINNA study, a randomized controlled trail of a nurse-led case management intervention with targeted recommendations in the elderly after MI in Germany. Reasons for non-adherence were described. Logistic mixed effects models and OLS (ordinary least squares) were used to analyze the effect of recommendations on the probability of adherence and the association between adherence and HRQoL.

**Results:**

One hundred and twenty-seven patients with 965 contacts were included. Frequent reasons for non-adherence to medication and recommendations were “forgotten” (22%; 11%), “reluctant” (18%; 18%), “side effects” (38%; 7%), “the problem disappeared” (6%; 13%), and “barriers” (0%; 13%). The probability of adherence was lowest for disease and self-management (38%) and highest for visits to the doctor (61%). Only if patients diverging from prescribed medication because of side effects were also considered as adherent, 3-year medication adherence was associated with a significant gain of 0.34 quality-adjusted life years (QALYs).

**Conclusions:**

Most important determinants of non-adherence to medication were side effects, and to recommendations reluctance. Recommended improvements in disease and self-management were least likely adhered. Medication adherence was associated with HRQoL.

**Trial registration:**

Current Controlled Trials ISRCTN02893746, retrospectively registered, date assigned 27/03/2009.

**Electronic supplementary material:**

The online version of this article (10.1186/s12877-018-0827-y) contains supplementary material, which is available to authorized users.

## Background

Coronary heart disease (CHD) is one of the leading causes of mortality and its’ incidence is likely to increase with increasing life expectancy [1]. It has been shown that patients with CHD who have suffered a myocardial infarction (MI) experienced significantly decreased health-related quality of life (HRQoL) compared with both, CHD patients without infarction and the general population, as measured by generic instruments such as EQ-5D-3 L and visual analog scale (VAS) [2, 3]. Despite adherence is deemed to be a necessary condition for effective therapy, empirical evidence shows insufficient observed and self-reported adherence to medication [4]. Besides adherence to medication, adherence to other aspects of therapy, such as healthy lifestyle, disease management and reduced personal risk factors are also important for effective therapy [5]. However, in health care provision, they have often been found to be poorly addressed [6]. Improving adherence to medication and healthy lifestyle is deemed to be important in heart diseases, especially after MI, because continuous therapy improves several clinical outcomes which are associated with improved HRQoL [7]. The association of adherence and HRQoL is less analyzed.

Case management programs are one approach to individualized secondary prevention. These programs are designed to improve adherence to medication and give recommendations to encourage patients to make healthy lifestyle choices, and optimal disease management [8]. It was found in previous research, that despite better knowledge, patients are often unable to implement healthy lifestyles into their lives [9]. This problem was addressed by randomized controlled trial (RCT) KORINNA, a nurse-led case management which was designed to analyze whether case management in elderly people with AMI can postpone unplanned readmission or death [10]. Previous evaluations showed that KORINNA can improve HRQoL in the elderly after MI but did not improve unplanned readmissions or death [11, 12]. It has not been extensively analyzed which determinants of adherence exist in the treatment group. There is some evidence about a positive association between adherence to medication and clinical outcomes in other settings [13–18]. But to the authors’ knowledge, there is little evidence about the impact of adherence in case-management interventions, especially with respect to nurses’ recommendations on HRQoL. Therefore, we analyzed this question in detail.

To improve adherence, its’ determinants need to be understood. There are some theoretical frameworks which suggest that the patient’s belief about the therapy’s effectiveness and costs have an impact on adherence [19]. Empirically, associations between patients’ characteristics and adherence were also studied. However, studies about patient reported reasons for non-adherence in heart diseases are rare [20, 21]. A recent study also calls for further research on patient-specific drivers for adherence to medication in post-MI patients [22]. This issue will also be assessed in this paper.

Increasing knowledge about non-adherence, its determinants, and effects on patient-relevant outcomes could help to address underlying problems, and to improve the effectiveness of case management interventions. Therefore, the first objective of this study was to identify determinants of non-adherence to both medication and recommendations in a case management intervention. The second objective was to verify whether adherence was associated with improved HRQoL.

## Methods

The data were obtained from the intervention group of the KORINNA RCT, which evaluated the effectiveness and cost-effectiveness of a case management intervention by trained nurses in elderly patients with an acute myocardial infarction.

### The KORINNA trial

The study design, intervention and results of the KORINNA trial are described elsewhere [10, 11, 23–25]. In short, 340 patients were recruited between September 2008 and May 2010. Recruited patients were 65+ years, and hospitalized as a result of MI at the Central Hospital of Augsburg, Germany. Enrollment and randomization took place between September 2008 and May 2010. Patients who lived in a nursing home or planned to move there, patients with severe comorbidities and a life expectancy of less than 1 year, or patients who were unable to communicate adequately in German were excluded. Retrospectively, 11 patients were excluded because they died or withdrew consent before hospital discharge. The trial was approved by the Ethics Committee of the Bavarian Chamber of Physicians (ISRCTN02893746).

### The case-management

The intervention was characterized by structured interviews carried out as home visits or telephone calls for up to 3 years after discharge from hospital. According to the specific needs, resources, and problems of each patient, nurses gave recommendations concerning healthy lifestyle and disease management. In the first year, an intervention contact was arranged at least every 3 months, and every 6 months in the second and third years. Adherence to recommendations and medication was evaluated only in the intervention group at each contact. Patients who did not adhere to medication or recommendation were asked for their reasons which were categorized. Further information with respect to categories, including examples are given in Additional file 1: Appendix A.

### Utilized sample for adherence evaluation

Because adherence could be only assessed in patients of the intervention group with at least two contacts with a nurse, other patients were excluded. Complementary and alternative therapies, and therapies or recommendations that could not be applied for objective reasons, such as readmission to hospital, were excluded. In the second analyses of the association of adherence and HRQoL only patients enrolled until the end of the trail or until death were included.

### Operationalization of medication and recommendation adherence

Included recommendations given by the study nurse concerned healthy lifestyle and were categorized by predefined classes and revised ex post. The urgency of recommendations with respect to the patient’s situation was judged by the nurse as “high”, “medium”, or “low”. Further information with respect to categories, including examples are given in Additional file 1: Appendix A.

Adherence to a single recommendation was defined as fully executing the advice. If a patient did not adhere to at least one highly urgent or two medium urgent recommendations of a contact, s/he was considered non-adherent at that contact until the next contact. Non-adherence to a medication application was defined if a patient had paused or discontinued therapy or changed the dose. Patients who had not taken one or more medication as prescribed were considered non-adherent at that contact. Otherwise, if non-adherence to medication was not identified, adherence was assumed.

If a patient was adherent for 80% of participation time, s/he was defined as 3-year adherent. This threshold is used to allow for some non-adherence which can be considered to be not problematic with respect to efficient therapy [26]. The 80% threshold is commonly used but somewhat arbitrary. Therefore, sensitivity analyses with 60 and 90% thresholds to adherence were done.

Non-adherence because of side effects in case of medication could be desirable, as they could be a sign of inappropriate therapy, or not desirable because there may be no better alternative. Because of this uncertainty, a second definition of medication adherence was used to analyze the association of adherence and HRQoL, where non-adherence to medication because of side effects was allowed.

### Potential determinants of non-adherence

Patient-reported reasons for non-adherence can be considered as causal drivers of adherence, if one assumes that patients answered honestly. Some recommendations might have been harder to adhere to than others. Changing nutrition for example might be more difficult than to make an appointment with the doctor. Therefore, besides patient-reported reasons, the type of recommendation was considered to be a potential driver of adherence.

### Operationalization of HRQoL

HRQoL was operationalized by VAS, where 100 represents perfect health and zero worst health possible [11]. It was assessed at each intervention contact. A case management intervention is a complex intervention which addresses multiple aspects in patients’ lives. Therefore, complex interventions require multi-dimensional outcome measurement like quality of life. In contrast to cardiovascular clinical outcomes like cardiovascular events, VAS-points reflect all patient relevant health problems including comorbidities. It is a measure of patient-reported health and is therefore considered to describe subjective health more appropriately than population-based valuation methods [27, 28]. A linear relationship was assumed between two VAS observations. QALYs were calculated as VAS-adjusted life years (VAS-ALs), as done in previous research [25]. In the event of death, a linear decline from the last observation to zero at the date of death was assumed. In a sensitivity analysis, we assumed the last observed VAS value to be constant until the event of death, because otherwise the timing of the last meeting could bias overall VAS-AL.

### Statistical methods

Simple descriptive statistics were used to present reasons for non-adherence, patients’ baseline characteristics and 3-year outcomes. Baseline values correspond to the first observation of a given variable. There were no missing values in baseline variables.

To analyze the impact of recommendation type on the probability of adherence, a generalized logistic mixed effects model with individual random intercept was used. In this type of model, the intercept is allowed to vary by individual. Patient specific intercepts are efficient to control for time-invariant heterogeneity within individuals in panel data, and therefore reduce the omitted variable bias [29]. The effect was controlled by recommendations’ urgency. Urgency is considered to be a potential confounder since it might be associated with both, the type of recommendation and the probability of adherence.

To follow the second objective, two models were used which were controlled by baseline-variables body mass index (BMI), New York Heart Association (NYHA) functional classification, age, gender, number of comorbidities and stressful events in follow-up.

First, a linear mixed effects model with random intercept, a variance component (VC) covariance structure and restricted maximum likelihood (REML) estimation was used. The VC covariance structure model allows each random effect to have a different variance. In contrast to maximum likelihood estimation the REML estimation is most appropriate for small sample sizes [30]. With this model the hypothesis that changing to adherence changes VAS was tested. Because of the risk of reversed causality, a lagged variable design was used which is appropriate to reduce that problem [31]. Accordingly, we estimated the effect of getting adherent to medication or a recommendation of a former contact on VAS at the next contact. Measures of adherence were operationalized as both, a fixed and a random effect. The fixed effects component is the variable of interest. It represents the effect of getting adherent. A random effect controls for the individual variance of this effect. Because of the random intercept, this model also controls for patient-specific, time-invariant heterogeneity. Additionally, the effect was controlled for days expired since randomization, as VAS and adherence were assumed to be affected by the passage of time.

Second, ordinary least squares (OLS) were used to test whether 3-year adherence was associated with overall VAS-AL gained. Because OLS is sensitive to outliers, a sensitivity analysis with least trimmed squares was used to perform an outlier robust estimation [32].

Although the problem of reversed causality was controlled by the lagged variable design, it could not be cut out completely. Therefore, the reversed relationship of adherence and VAS was analyzed in a sensitivity analysis, where the effect of VAS at one intervention contact on adherence until the next contact was also estimated. This analysis is presented in Additional file 1: Appendix B. Because the results were not significant, risk of reversed causality was assumed to be small.

All statistical analyses were conducted in SAS 9.3.

## Results

Of 161 patients from the KORINNA intervention group 127 patients were included. Thirty-four patients were excluded because they never had an intervention contact. From the analyses of 3-year adherence, 11 patients were excluded because they left the trial before the end for reasons other than death. Baseline characteristics and 3-year outcomes are presented in Table 1.

**Table 1:** Patient baseline characteristics and 3-year outcomes

*N* = 127	N/mean	%/SD
Baseline characteristics		
Male (n, %)	81	63.8
Diabetes (n, %)	34	26.8
CHF (n, %)	34	26.8
Neither diabetes nor CHF (n, %)	72	56.7
Diabetes or CHF (n, %)	42	33.1
Diabetes and CHF (n, %)	13	10.2
Age (mean, SD)	74.7	5.7
VAS (mean, SD)	63.3	17.9
BMI (mean, SD)	27.0	4.0
3-year outcomes (*N* = 116)		
VAS-AL (mean/SD)	1.82	0.62
Adherent to medications (n, %)^a^	101	87.1
Adherent to recommendations (n, %)^a^	79	68.1
Adherent to medications and recommendations (n, %)^a^	72	62.1
Non-adherent to medications and recommendations (n, %)^a^	8	6.9
Non-adherent to either medications or recommendations (n, %)^a^	36	31.0

^a^Adherence refers to 3-year adherence

*BMI* body mass index, *CHF* chronic heart failure, *N* number of patients, *SD* standard deviation; *VAS* visual analog scale, *VAS-AL* VAS-adjusted life years

Patients were mostly male and between 65 and 91 years old. About 43% suffered from comorbidities, diabetes, congestive heart failure, or both. Three-year adherence to medication was higher than to recommendations (87% vs. 68%). Within 3 years, patients achieved 1.82 VAS-ALs (min. 0.07, max. 2.81). The sensitivity analysis with fixed VAS until death affected three patients and did not change overall results (results are not reported).

### Determinants of medication adherence

Within 3 years, 127 included patients received 965 intervention contacts. Non-adherence to prescribed medication was observed in 72 contacts. Of those, a changed dose (33%) or paused medication (32%) was observed most frequently. Side effects, the most important reason for non-adherence to medication, mostly led to discontinuations (44%) or changed doses (37%). Forgetfulness, the second most frequently reported reason, was mostly associated with paused therapies (94%). Reluctant patients changed their dose in 54% of cases (Additional file 1: Appendix Table D1, C1).

### Determinants of recommendation adherence

For 771 of 889 recommendations adherence was assessed. Eighty recommendations concerned natural therapies and were excluded. Finally 691 were included in the analysis. Thereof, 314 were not fully adhered to (Additional file 1: Appendix Table C2).

Mostly patients did not report a clear reason for non-adherence (21%). Of evaluable reasons, reluctance was the most frequent, followed by disappearance of the underlying problem and barriers. In case of recommended doctor visits disappearance of the underlying problem (21%), forgetfulness (15%), and postponed visits (14%) were most frequently reported. In case of recommended improvements in control of vital signs and blood glucose, reluctance (39%) and forgetfulness (17%) were the major reasons (details in Additional file 1: Appendix Table C2). Adjusted results from logistic estimations showed that the probability of adherence was highest if visits to the doctor were recommended (61%), and lowest in case of disease and self-management (38%), nutrition (40%), and control of vital signs and blood glucose (43%) (Table 2).

**Table 2:** Generalized logistic mixed effects model – the effect of recommendation types and urgency on adherence

*N* = 686 (110 patients)	Adherence to recommendations					
Variable	OR	95% CI limits		Probability of adherence in %	95% CI limits	
Type of recommendation (ref = visit to the doctor)						
Others	0.55	0.25	1.21	46.25	27.82	65.77
Disease and self-management	0.39	0.17	0.88	37.69	21.48	57.21
Mobility and fall prevention	0.53	0.25	1.11	45.22	27.91	63.77
Control of vital signs and blood glucose	0.49	0.31	0.77	43.42	31.56	56.08
Nutrition	0.42	0.24	0.72	39.66	28.25	52.33
Visit to the doctor				61.06	51.41	69.92
Urgency (ref = low)						
High	1.87	0.80	4.36	50.59	42.67	58.47
Medium	1.90	0.78	4.58	50.95	40.97	60.86
Low				35.41	19.54	55.30

*CI* confidence interval, *OR* odds ratio, *ref* reference

### Adherence and HRQoL

Three-year adherence to recommendations or medication was not significantly associated with VAS-AL (− 0.06, *p* = 0.52; 0.21, *p* = 0.13). Becoming adherent to recommendations or medication did not cause significant changes in VAS points (− 2.04, *p* = 0.11; − 1.47, *p* = 0.4) (Additional file 1: Appendix Table D1). Significant effects were only found if adherent patients included patients who diverged from prescribed therapy because of side effects. In this case, in 3-year medication adherent patients HRQoL increased by 0.40 VAS-AL (Table 3, details in Additional file 1: Appendix Table D1).

**Table 3:** Results of OLS estimation: Association of adherence and VAS-AL

*N* = 116 patients	VAS-AL			VAS-AL (adherent in presence of side effects)		
3-year adherence	Estimate	SE	Pr > |t|	Estimate	SE	Pr > |t|
Adherence to medication	0.21	0.14	0.1263	0.40	0.18	0.0270
Adherence to recommendations	−0.06	0.10	0.5159	−0.08	0.10	0.4033

All results are controlled for baseline variables (BMI, NYHA, Age, gender, number of comorbidities) and stressful event in follow-up

*BMI* body mass index, *NYHA* classification of heart failure of the New York Heart Association, *SE* standard error, *VAS* visual analog scale, *VAS-AL* VAS-adjusted life years, *LME* linear mixed effects

Sensitivity analyses with 60 and 90% thresholds to adherence did not show significant results (Additional file 1: Appendix Table D2). Outlier robust estimations barely changed estimates. The significant association of medication adherence and VAS-AL remained but decreased to 0.32 VAS-AL (Additional file 1: Appendix Table D3).

## Discussion

To increase the effectiveness of case management interventions, adherence to case manager’s recommendations and to prescribed medication is deemed to be important. To the authors’ knowledge, the role of adherence in case management interventions with respect to HRQoL and reasons for non-adherence are not fully understood and rarely studied. Based on the intervention group in the KORINNA trial, determinants of non-adherence to medication and recommendations in patients after MI were identified first. Second, it was verified whether increased adherence was associated with improved HRQoL in those patients.

### Determinants of adherence

It was found that side effects, forgetfulness, and being reluctant were the most important reasons for non-adherence to medication. Patients with side effects mostly discontinued therapy or changed the dose. On the one hand, this could have been the right decision and in concordance with the prescribing physician. On the other hand, it could be hazardous. Based on the available data, this could not be evaluated. Forgetfulness was more often associated with paused applications than with discontinuation, and never with a change in dose. Therefore, patients sometimes forgot the application rather than the correct dosage. Reluctance was more often associated with changed doses than with discontinuation or other kinds of non-adherence. This implies that patients were usually convinced about the medication’s necessity, but did not believe in the necessity of prescribed dosing.

Reasons for non-adherence to recommendations differed between the different types of recommendations. Mostly, visits to the doctor, improvements in nutrition, and improved control of vital signs and blood glucose were recommended. In case of non-adherence to doctor’s appointments, the most frequent reason was the disappearance of the underlying problem. This might be problematic if abating symptoms, such as high blood glucose levels or angina pectoris, should still be treated [7]. The most important reason for non-adherence to improvements in control of vital signs and blood glucose was being reluctant, which can be problematic especially in post-MI patients [7]. Adherence to improvements in nutrition is usually considered to be important. For example achieving body fat goals is recommended in MI patients [7]. The finding that non-adherence to nutrition was mostly due to barriers is in line with previous research which found that patients find it hard to adhere to diet restrictions [33].

It was found that patients were more likely to visit a doctor than to improve self- and disease management, nutrition, or control of vital signs and blood glucose. It was also found that 3-year adherence to medication was more frequent than to recommendations. Furthermore, non-adherence and reluctance were bigger problems in recommendations than in medications. Therefore, the findings of this research support existing evidence that patients have greater belief in medical therapy than in healthy lifestyle and disease management-related recommendations [33]. They also suggest that patients have more problems in explaining their reasons for non-adherence to recommendations than to medication, because patients did not report specific reasons for non-adherence to recommendations more frequently than to medication.

### The association of adherence and HRQoL

Results about the effect of adherence on HRQoL were sensitive to the definition of adherence. With the first definition, significant effects of adherence on HRQoL could not be identified. With the second definition of medication adherence (side effects were allowed) a significant increase of 0.34 VAS-AL was found in patients who were 80% of 3-year follow up adherent to medication. The result has shown to be robust to outliers. This is some evidence for the conclusion that non-adherence because of side effects was desirable in the KORINNA trail. However, this finding is of limited generalizability because the study population was educated by the study-nurses and can therefore be assumed to be more qualified for autonomous therapy decisions than the general population [10]. An explanation for the positive effect of medication adherence, as defined in the second definition, on VAS-AL but not on VAS is the consideration of long-term effects and length of periods in different health states. We could not find significant effects of adherence to recommendations on VAS or VAS-AL. There are several explanations possible. First, the recommendations’ quality was inadequate to improve health. However, nurses had a special evidence-based training for post-MI care, which should have insured high quality. It was found that adherence to medication but not to recommendations showed significant effects. This might indicate that in contrast to recommendations, medication was more effective with respect to VAS-AL. Second, there might have been ceiling effects in recommendation and medication adherence, as the study population comprised only the intervention group and the intervention was designed to guarantee high adherence. Third, the power in the intervention group might not have been big enough to identify significant effects. Fourth, the threshold of 80% might not comply with the true critical level of non-adherence. Critical levels of adherence are rarely studied [34]. Significant estimates were only found in the primary analysis but not in sensitivity analyses with 60 and 90% thresholds to adherence. Therefore, the 80% threshold seems to be valid to identify significant effects in the KORINNA’s intervention group. Besides, the utilized definition of medication adherence is similar to existing research. Furthermore, recommendations were only necessary if there was some kind of non-optimal behavior. Therefore, recommendations are to some degree already an indicator of non-adherence. Lastly, even though additional analyses about the effect of VAS on adherence did not show significant results, reversed causality cannot be ruled out.

Because of the single-center design of the KORINNA trial, the findings are of limited generalizability. Because a very specific intervention was analyzed, the results might only apply to patients after MI in similar interventions. An advantage of this study is the panel data, which allowed controlling for unobserved, time-invariant heterogeneity in the mixed effects models. Panel data considered to be more valid to identify causal associations than cross-sectional data, as often utilized in surveys concerning the analysis of adherence [35]. Another advantage is the huge number of interviews and the prospective study design. Despite the problems discussed, this study contributes to the limited existing evidence about determinants of non-adherence and effects of adherence, especially to recommendations.

## Conclusions

Patient-reported reasons for non-adherence and recommendation types were considered to be determinants of adherence. It was found that reasons for non-adherence differed with type of therapy (healthy lifestyle vs. medication) and type of recommendations with respect to healthy lifestyle. We found different reasons for non-adherence. For example reluctance (side effects) was the most important for non-adherence recommendations (medication). We also found that different recommendations were associated with different risks of non-adherence. The greatest risk of non-adherence to recommendations was associated with disease and self-management, visits no the doctor was significantly more likely to be adhered. Knowing about these determinants of non-adherence will help case-managers to take appropriate and stratified precautions to prevent non-adherence in dependence of the type of therapy.

The effect of adherence on HRQoL was sensitive to the definition of adherence. Patients who were adherent to medication within 3 years only gained 0.34 VAS-AL if non-adherence because of side effects was allowed. Therefore, it can be concluded that non-adherence to medication because of side effects was beneficial in the analyzed study population. However, the intervention group was schooled and can therefore be considered to be more qualified for therapy decisions than the general population.

## Additional file

Additional file 1: **Appendix A.** Categories of recommendations and reasons for non-adherence. This file describes the definition of categories of recommendations (Table A1) and of the categories of reasons for non-adherence (Table A2). **Appendix B.** Impact of VAS on adherence. This file motivates and describes methods used for the analysis of reversed causality between adherence and VAS. Table B1 presents results from the logistic mixed effects model. **Appendix C.** Reasons for non-adherence. This file presents descriptive statistics about the reasons for non-adherence. Table C1 relates reasons for non-adherence to types of non-adherence. Table C2 relates reasons for non-adherence to recommendation types. **Appendix D.** The association of adherence and HRQoL. This file presents detailed results of the primary analyses of the association of adherence and VAS and VAS-AL (Table D1). Table D2 summarizes sensitivity analyses with varying thresholds to adherence. Table D3 summarizes sensitivity analyses with outlier robust least trimmed squares estimations. (DOCX 48 kb)

## Abbreviations

BMI: Body mass index
CHD: Coronary heart disease
HRQOL: Health related quality of life
MI: Myocardial infarction
NYHA: New York Heart Association
OLS: Ordinary least squares
QALYs: Quality-adjusted life years
REML: Restricted maximum likelihood
VAS: Visual analog scale
VAS-ALs: VAS-adjusted life years
VC: Variance components
