# Supplementary material for: Determinants of adherence and effects on health-related quality of life after myocardial infarction: a prospective cohort study
Source: BMC Geriatr. 2018 Jun 7;18:136. doi: 10.1186/s12877-018-0827-y (PMC6001009; doi:10.1186/s12877-018-0827-y)
Supplement: Supplementary file 1 — Appendix A. Categories of recommendations and reasons for non-adherence. This file describes the definition of categories of recommendations (Table A1) and of the categories of reasons for non-adherence (Table A2). Appendix B. Impact of VAS on adherence. This file motivates and describes methods used for the analysis of reversed causality between adherence and VAS. Table B1 presents results from the logistic mixed effects model. Appendix C. Reasons for non-adherence. This file presents descriptive statistics about the reasons for non-adherence. Table C1 relates reasons for non-adherence to types of non-adherence. Table C2 relates reasons for non-adherence to recommendation types. Appendix D. The association of adherence and HRQoL. This file presents detailed results of the primary analyses of the association of adherence and VAS and VAS-AL (Table D1). Table D2 summarizes sensitivity analyses with varying thresholds to adherence. Table D3 summarizes sensitivity analyses with outlier robust least trimmed squares estimations. (DOCX 48 kb) [file 12877_2018_827_MOESM1_ESM.docx]

# Additional file 1 Appendices

## A Categories of recommendations and reasons for non-adherence

### Table A1: description of categories of recommendations

| Recommendation | Description of category |
| --- | --- |
| Visit to the doctor | A visit to the doctor was recommended, for example because of worsening outcomes or symptoms. |
| Nutrition | Any recommendation with respect to nutrition. For example, nurses recommended eating less or more healthily, and drinking more. |
| Control of vital signs and blood glucose | Any advice to improve monitoring, for example more regular control of blood glucose and blood pressure. |
| Mobility and fall prevention | Any advice with the aim of preventing falls or improving mobility. For example, it was recommended to avoid tripping hazards, to exercise regularly, or to visit a heart sports group. |
| Disease and self‑management | Any recommendation to improve disease outcomes or to manage related organizational issues, which are not covered by “visits to the doctor”, “nutrition”, “control of vital signs and blood glucose”, or “mobility and fall prevention”. Examples include recommendations about how to get important information that cannot be provided by the study nurse or to wear compression socks regularly. |
| Others | All other recommendations that are not covered by defined categories. |

### Table A2: description of categories of reasons for non-adherence

| Reason for non‑adherence | Description of category |
| --- | --- |
| No clear reason given | There is either no reason given or the patient did not answer with a clear reason for non-adherence. |
| Forgotten | The patient reported that s/he had forgotten the recommendation or medication application. |
| No time | The patient reported that s/he did not find the time to conduct the recommendation or take the medication. |
| Reluctant | The patient showed that s/he did not adhere because s/he thought it was unnecessary. |
| Side effects or inconveniences | The patient revealed that s/he did not adhere to a recommendation or to his/her medication because it was associated with inconveniences or similar disutility. For example, one patient did not adhere to the recommendation to join a sports group because the weather was bad. Another example was discontinuation of medication application because of tachycardia or other side effects. |
| Choice of an alternative action | For example, the patient did not conduct the recommendation to solve an underlying problem, but chose an alternative. |
| The problem disappeared | The underlying issue resolved on its own. The problem that caused the nurse to recommend some action resolved, or symptoms disappeared. One example would be a patient who paused lipid-lowering drugs because his/her cholesterol levels improved. |
| External influences | “External influences” comprise reasons where a patient did not adhere because of other information, for example from other people or literature. For example, one patient did not take a cholesterol-lowering drug because s/he read a book that doubted the risk of high cholesterol. |
| Barriers | “Barriers” relate to unspecific reasons that prevented the patient from adhering even though s/he wanted to. For example, some patients tried but did not manage to eat less, drink more, or stop smoking. |
| Postponed | Recommendations are “postponed” if a patient emphasized his/her will to adhere but postponed the execution until another point in time. |
| Other reasons | Rarely reported reasons (<3) |

## B Impact of VAS on adherence

Theory suggests that not only does adherence improve health, but also that perceived HRQoL could have an impact on adherence. This can be derived from the health belief model, which suggests that adherence is determined from the perceived threat of adverse health effects [1, 2]. Perceived threat might be determined by worsening HRQoL. Therefore, decreased HRQoL at one point in time might have an impact on the probability of being adherent during the next period, which has a positive effect on health outcomes at the end of that period. To deal with this problem of reversed causality, we took advantage of the longitudinal data and tested the significance of the reversed direction of effect. This was done by estimating the effect of increased HRQoL at one point in time on the probability of adherence in the next period. Following this approach, we used two logistic mixed effects models with random intercepts. Dependent variables were either medication adherence or recommendation adherence. The independent variable in both models was perceived health, measured as VAS. This effect is controlled by potential confounding variables such as time expired since randomization and baseline variables, e.g., diagnoses of diabetes or chronic heart failure (CHF), NYHA stadium, body mass index (BMI), age, and gender. Estimates were transformed to odds ratios.

The estimations of the logistic mixed models of HRQoL on both medication and recommendation adherence are presented in Table B1. In our sample, a 1-point increase in VAS was associated with increased chance of being adherent to medication; a 1-point VAS increase was also associated with decreased chance of being adherent to recommendations (OR=0.991). However, these effects were not statistically significant.

### Table B1: Logistic mixed effects models—the effect of VAS on adherence to recommendations and medications

| N=965 (127 patients) | Adherence to medications | | | Adherence to recommendations | | |
| --- | --- | --- | --- | --- | --- | --- |
| Variable | OR | 95% CI limits | | OR | 95% CI limits | |
| Binary variables |  |  |  |  |  |  |
| Male (ref = female) | 0.991 | 0.456 | 2.153 | 1.656 | 0.913 | 3.005 |
| Continuous variables (unit) |  |  |  |  |  |  |
| Number of comorbidities | 1.509 | 0.849 | 2.679 | 1.156 | 0.736 | 1.815 |
| Age baseline (years) | 0.945 | 0.883 | 1.011 | 1.003 | 0.951 | 1.058 |
| NYHA baseline (class) | 0.706 | 0.252 | 1.978 | 0.726 | 0.319 | 1.651 |
| BMI baseline (point) | 0.963 | 0.878 | 1.055 | 0.941 | 0.877 | 1.009 |
| VAS (point) | 1.014 | 0.996 | 1.033 | 0.991 | 0.977 | 1.005 |
| Days expired since randomization | 1.000 | 1.000 | 1.001 | 1.002 | 1.001 | 1.002 |
| BMI, body mass index; CHF, chronic heart failure; CI, confidence interval; DM, diabetes mellitus; N, number of observations; NYHA, New York Heart Association; OR, odds ratio; ref, reference; VAS, visual analog scale. | | | | | | |

## C Reasons for non-adherence

### Table C1: Reasons for and types of non-adherence to medication

| Reason for non-adherence | Type of non-adherence | | | | | | | | Total | |
| --- | --- | --- | --- | --- | --- | --- | --- | --- | --- | --- |
|  | Changed dose | | Changed time of application | | Discontinued | | Paused | |  |  |
|  | n | %^a^ | n | %^a^ | n | %^a^ | n | %^a^ | n | %^b^ |
| No clear reason given | 0 | 0.0 | 1 | 100.0 | 0 | 0.0 | 0 | 0.0 | 1 | 1.4 |
| Forgotten | 0 | 0.0 | 1 | 6.2 | 0 | 0.0 | 15 | 93.8 | 16 | 22.2 |
| No time | 0 | 0.0 | 0 | 0.0 | 0 | 0.0 | 0 | 0.0 | 0 | 0 |
| Reluctant | 7 | 53.9 | 3 | 23.1 | 3 | 23.1 | 0 | 0.0 | 13 | 18.1 |
| Side effects or inconveniences | 10 | 37.1 | 0 | 0.0 | 12 | 44.4 | 5 | 18.5 | 27 | 37.5 |
| Choice of an alternative | 0 | 0.0 | 0 | 0.0 | 0 | 0.0 | 1 | 100.0 | 1 | 1.4 |
| The problem disappeared | 1 | 25.0 | 0 | 0.0 | 3 | 75.0 | 0 | 0.0 | 4 | 5.6 |
| External influences | 1 | 20.0 | 0 | 0.0 | 2 | 40.0 | 2 | 40.0 | 5 | 6.9 |
| Barriers | 0 | 0.0 | 0 | 0.0 | 0 | 0.0 | 0 | 0.0 | 0 | 0.0 |
| Postponed | 0 | 0.0 | 0 | 0.0 | 0 | 0.0 | 0 | 0.0 | 0 | 0.0 |
| Other reasons | 5 | 100.0 | 0 | 0.0 | 0 | 0.0 | 0 | 0.0 | 5 | 6.9 |
| Total | 24 | 33.3 | 5 | 6.9 | 20 | 27.8 | 23 | 31.9 | 72 | 100.0 |
| ^a^ Row percentage ^b^ Column percentage | | | | | | | | | | |

### Table C2: Reasons for non‑adherence to recommendation types

| Recommendation type | Visit to the doctor | | Nutrition | | Control of vital signs and blood glucose | | Mobility and fall prevention | | Disease and self-management | | Other | | Total |  |
| --- | --- | --- | --- | --- | --- | --- | --- | --- | --- | --- | --- | --- | --- | --- |
|  | N | %^a^ | N | %^a^ | N | %^a^ | N | %^a^ | N | %^a^ | N | %^a^ | N | %^a^ |
| Total | 387 | 56.0 | 82 | 11.9 | 123 | 17.8 | 35 | 5.1 | 32 | 4.6 | 32 | 4.6 | 691 | 100.0 |
| Urgency |  |  |  |  |  |  |  |  |  |  |  |  |  |  |
| High | 315 | 81.4 | 45 | 54.9 | 89 | 72.4 | 17 | 48.6 | 15 | 46.9 | 22 | 68.8 | 503 | 72.8 |
| Medium | 66 | 17.1 | 21 | 25.6 | 32 | 26.0 | 16 | 45.7 | 8 | 25.0 | 7 | 21.9 | 150 | 21.7 |
| Low | 4 | 1.0 | 16 | 19.5 | 1 | 0.8 | 2 | 5.7 | 7 | 21.9 | 3 | 9.4 | 33 | 4.8 |
| Missing | 2 | 0.5 | 0 | 0.0 | 1 | 0.8 | 0 | 0.0 | 2 | 6.3 | 0 | 0.0 | 5 | 0.7 |
| Non‑adherence | 141 | 36.4 | 47 | 57.3 | 72 | 58.5 | 18 | 51.4 | 19 | 59.4 | 17 | 53.1 | 314 | 100.0 |
| Reasons for non-adherence | | | | | | | | | | | | | | |
| No clear reason given | 26 | 18.4 | 13 | 27.7 | 18 | 25.0 | 2 | 11.1 | 3 | 15.8 | 3 | 17.7 | 65 | 20.7 |
| Forgotten | 21 | 14.9 | 2 | 4.3 | 12 | 16.7 | 0 | 0.0 | 0 | 0.0 | 1 | 5.9 | 36 | 11.5 |
| No time | 7 | 5.0 | 0 | 0.0 | 1 | 1.4 | 0 | 0.0 | 0 | 0.0 | 0 | 0.0 | 8 | 2.6 |
| Reluctant | 12 | 8.5 | 3 | 6.4 | 28 | 38.9 | 6 | 33.3 | 3 | 15.8 | 5 | 29.4 | 57 | 18.2 |
| Side effects or inconveniences | 16 | 11.4 | 2 | 4.3 | 2 | 2.8 | 1 | 5.6 | 1 | 5.3 | 1 | 5.9 | 23 | 7.3 |
| Choice of an alternative | 0 | 0.0 | 4 | 8.5 | 2 | 2.8 | 3 | 16.7 | 1 | 5.3 | 2 | 11.8 | 12 | 3.8 |
| The problem disappeared | 29 | 20.6 | 5 | 10.6 | 1 | 1.4 | 0 | 0.0 | 4 | 21.1 | 2 | 11.8 | 41 | 13.1 |
| External influences | 3 | 2.1 | 0 | 0.0 | 0 | 0.0 | 0 | 0.0 | 0 | 0.0 | 0 | 0.0 | 3 | 1.0 |
| Barriers | 8 | 5.7 | 18 | 38.3 | 3 | 4.2 | 5 | 27.8 | 5 | 26.3 | 1 | 5.9 | 40 | 12.7 |
| Postponed | 19 | 13.5 | 0 | 0.0 | 4 | 5.6 | 1 | 5.6 | 2 | 10.5 | 0 | 0.0 | 26 | 8.3 |
| Other reasons | 0 | 0.0 | 0 | 0.0 | 1 | 1.4 | 0 | 0.0 | 0 | 0.0 | 2 | 11.8 | 3 | 1.00 |
| ^a^ Column percentage |  | | | | | | | | | | | | | |

## D The association of adherence and HRQoL

### Table D1: The effect of adherence on VAS and VAS-AL

| OLS: N=116 patients  LME: N=965  (127 patients) | Linear OLS model | | | | | | Linear mixed effects model | | | | | |
| --- | --- | --- | --- | --- | --- | --- | --- | --- | --- | --- | --- | --- |
|  | VAS-AL | | | VAS-AL (without side effects) | | | VAS | | | VAS (without side effects) | | |
| Variable | estimate | SE | Pr > \|t\| | estimate | SE | Pr > \|t\| | estimate | SE | Pr > \|t\| | estimate | SE | Pr > \|t\| |
| Intercept | 5.01 | 0.74 | <0.0001 | 5.00 | 0.73 | <0.0001 | 177.71 | 16.58 | <0.0001 | 177.92 | 16.69 | <0.0001 |
| Adherence to medication |  |  |  |  |  |  |  |  |  |  |  |  |
| 3-year adherence | 0.21 | 0.14 | 0.1263 | 0.40 | 0.18 | 0.0270 |  |  |  |  |  |  |
| Contact adherence |  |  |  |  |  |  | –1.47 | 1.73 | 0.3994 | –2.48 | 2.20 | 0.2697 |
| Adherence to recommendations |  |  |  |  |  |  |  |  |  |  |  |  |
| 3-year adherence | –0.06 | 0.10 | 0.5159 | –0.08 | 0.10 | 0.4033 |  |  |  |  |  |  |
| Contact adherence |  |  |  |  |  |  | –2.04 | 1.24 | 0.1061 | –2.03 | 1.24 | 0.1068 |
| Time expired since randomization | / | / | / | / | / | / | –0.001 | 0.001 | 0.6244 | –0.001 | 0.001 | 0.6053 |
| BMI baseline | 0.002 | 0.01 | 0.8392 | 0.001 | 0.01 | 0.9206 | –0.56 | 0.26 | 0.0337 | –0.56 | 0.26 | 0.0358 |
| NYHA baseline | –0.36 | 0.16 | 0.0219 | –0.35 | 0.15 | 0.0222 | –5.10 | 3.15 | 0.1058 | –5.07 | 3.15 | 0.1083 |
| Age baseline | –0.04 | 0.01 | <0.0001 | –0.04 | 0.01 | <0.0001 | –1.16 | 0.19 | <0.0001 | –1.15 | 0.19 | <0.0001 |
| Male | 0.23 | 0.10 | 0.0211 | 0.23 | 0.10 | 0.0228 | 6.65 | 2.21 | 0.0027 | 6.60 | 2.21 | 0.0030 |
| Number of comorbidities baseline | –0.17 | 0.07 | 0.0179 | –0.19 | 0.07 | 0.0094 | –3.89 | 1.61 | 0.0159 | –4.01 | 1.61 | 0.0132 |
| Stressful event in follow-up | –0.77 | 0.26 | 0.0035 | –0.85 | 0.26 | 0.0014 | –1.40 | 1.12 | 0.22 | –1.35 | 1.12 | 0.2297 |
| Adjusted R-squared | 0.42 |  |  | 0.44 |  |  |  |  |  |  |  |  |
| BMI, body mass index; NYHA, classification of heart failure of the New York Heart Association; SE, standard error; VAS, visual analog scale; VAS-AL, VAS-adjusted life years; LME, linear mixed effects.  All results are controlled for baseline variables (BMI, NYHA, age, gender, number of comorbidities) and stressful events in follow-up. Furthermore, the LME models are controlled for time expired since randomization. | | | | | | | | | | | | |

### Table D2: Sensitivity analyses: the effect of adherence on VAS-AL, 60% and 90% adherence threshold

| OLS: N=116 patients | 60% adherence threshold^a^ | | | | | | 90% adherence threshold^b^ | | | | | |
| --- | --- | --- | --- | --- | --- | --- | --- | --- | --- | --- | --- | --- |
|  | VAS-AL | | | VAS-AL (without side effects) | | | VAS | | | VAS (without side effects) | | |
| Variable | estimate | SE | Pr > \|t\| | estimate | SE | Pr > \|t\| | estimate | SE | Pr > \|t\| | estimate | SE | Pr > \|t\| |
| Intercept | 5.02 | 0.76 | <0.0001 | 5.15 | 0.86 | <0.0001 | 5.00 | 0.73 | <0.0001 | 5.02 | 0.73 | <0.0001 |
| Adherence to medication (3 years) | 0.15 | 0.18 | 0.4191 | 0.04 | 0.35 | 0.9084 | 0.19 | 0.11 | 0.1069 | 0.21 | 0.14 | 0.1206 |
| Adherence to recommendations  (3 years) | 0.01 | 0.15 | 0.9715 | –0.002 | 0.15 | 0.9883 | –0.04 | 0.09 | 0.6430 | –0.04 | 0.09 | 0.6970 |
| Adjusted R-squared | 0.41 |  |  | 0.41 |  |  | 0.42 |  |  | 0.42 |  |  |
| VAS, visual analog scale; VAS-AL, VAS-adjusted life years.  All analyses are controlled for the occurrence of a stressful event in follow-up and baseline variables (BMI, NYHA, age, gender, number of comorbidities).  ^a^ Adherent to recommendations: 102 patients; adherent to medication: 108 patients  ^b^ Adherent to recommendations: 63 patients; adherent to medication: 91 patients | | | | | | | | | | | | |

### Table D3: Sensitivity analyses: the association of adherence and VAS-AL with outlier robust least trimmed squares (LTS)

| OLS: N=116 patients | 80% adherence threshold | | | | | |
| --- | --- | --- | --- | --- | --- | --- |
|  | VAS-AL (76.72% of observations used) | | | VAS-AL (without side effects) (81.03% of observations used) | | |
| Variable | estimate | SE | Pr > \|t\| | estimate | SE | Pr > \|t\| |
| Intercept | 4.72 | 0.60 | <0.0001 | 4.16 | 0.61 | <0.0001 |
| Adherence to medication (3 years) | –0.02 | 0.11 | 0.8684 | 0.32 | 0.15 | 0.0308 |
| Adherence to recommendations (3 years) | –0.03 | 0.07 | 0.6472 | –0.07 | 0.07 | 0.3470 |
| R-squared | 0.48 |  |  | 0.51 |  |  |
| VAS, visual analog scale; VAS-AL, VAS-adjusted life years.  All analyses are controlled for the occurrence of a stressful event in follow-up and baseline variables (BMI, NYHA, age, gender, number of comorbidities). | | | | | | |

## Appendices Literature

1. Rosenstock IM: Why people use health services. Milbank Mem Fund Q 1966, 44(3):Suppl:94-127.

2. Becker MH, Maiman LA: Sociobehavioral determinants of compliance with health and medical care recommendations. Med Care 1975, 13(1):10-24.
